# Mitochondrial DNA copy number as a biomarker of oxidative stress and inflammation in premature ovarian insufficiency

**DOI:** 10.3389/fmed.2026.1719451

**Published:** 2026-08-05

**Authors:** Shufang Xiao, Meixian Zhu

**Affiliations:** Department of Obstetrics and Gynecology, First Hospital of Putian City, Putian, Fujian, China

**Keywords:** biomarker, chronic inflammatory response, mitochondrial DNA copy number, oxidative stress response, premature ovarian insufficiency

## Abstract

To examine how mitochondrial DNA copy number (mtDNAcn) relates to oxidative stress and persistent inflammation in individuals with premature ovarian insufficiency (POI). A single-center case-control investigation, spanning January 2020 to December 2022, recruited 100 POI patients alongside 100 healthy volunteers at our institution. We evaluated baseline demographic and clinical traits across both cohorts. Using qPCR, we quantified mtDNAcn in peripheral blood, while ELISA measured serum markers of oxidative stress (reactive oxygen species [ROS], superoxide dismutase [SOD], glutathione S-transferase [GST]) and inflammatory cytokines (tumor necrosis factor-α [TNF-α], interferon-γ [IFN-γ], interleukin-1β [IL-1β], interleukin-10 [IL-10], transforming growth factor-β [TGF-β]). We applied ROC curve analysis to gauge mtDNAcn’s diagnostic potential for POI, with Pearson correlation and multiple linear regression exploring links to oxidative and inflammatory factors. No notable differences emerged in age, body mass index, blood pressure, parity, gravidity, or menarche onset (*P* > 0.05). POI patients displayed elevated serum TNF-α, IFN-γ, IL-1β, and ROS, but reduced IL-10, TGF-β, SOD, GST, and mtDNAcn (*P* < 0.05). Exploratory ROC analysis suggested that mtDNAcn had good predictive capacity in this single-center cohort (AUC = 0.951, 95% CI: 0.922–0.980). mtDNAcn positively associated with IL-10, TGF-β, SOD, and GST, yet inversely with TNF-α, IFN-γ, IL-1β, and ROS (*P* < 0.05). Multiple linear regression identified these markers as independently associated with mtDNAcn. Lower mtDNAcn in peripheral blood of POI patients was associated with increased oxidative stress and chronic inflammation. mtDNAcn may serve as a potential non-invasive biomarker candidate for POI, but prospective studies with external validation are required to establish its clinical utility.

## Introduction

Declining ovarian function is an important sign of female aging, and women usually start to experience menstrual loss, amenorrhea, dryness, palpitations, and insomnia around the age of 45, which is a natural process for the body to adapt to the decline of ovarian function. Premature ovarian insufficiency (POI) is the term for ovarian failure symptoms that some women have before the age of 40 due to a variety of reasons, including metabolic abnormalities, gonadotropin malfunction, immune system damage, chemical damage, hereditary factors, and lifestyle choices (1, 2). POI can cause a series of health problems, including fertility decline or loss, hot flashes, osteoporosis, cardiovascular disease, sexual dysfunction, etc., which seriously affect the physical and mental health of patients. With the in-depth study of POI, some scholars have found that mitochondria are among the most important organelles in oocytes, and mitochondrial dysfunction is the most important reason for the decline of oocyte quality and embryo development potential (3, 4). Some studies have also pointed out that mitochondrial DNA copy number (mtDNAcn) can be used as a biological indicator to predict oocyte quality and embryo development potential, autologous homologous mitochondrial transfer and the use of mitochondrial nutrients can be used as a treatment option (5) for patients with POI and insufficient ovarian reserve.

The imbalance of Th1/Th2 cytokines can cause ovarian tissue to be in a chronic inflammatory state. Inflammatory stimulation can destroy follicular development and promote oocyte apoptosis, leading to ovarian tissue damage and ovarian function decline (6). In addition, persistent inflammation can create a high number of reactive oxygen species (ROS), superoxide dismutase (SOD), glutathione S-transferase (GST) and other chemicals, resulting in oxidative stress damage. With the accumulation of oxidative stress, egg destruction, follicle atresia and ovarian tissue fibrosis will be enhanced, and the likelihood of ovarian function decline will be greatly raised (7). In conclusion, POI is a typical condition of ovarian function decline, and chronic inflammation and oxidative stress are connected to the occurrence and progression of ovarian function decline. The relationship between mtDNAcn, oxidative stress, and inflammation in POI remains uncertain. Based on this, this study examined the alterations in mtDNAcn in POI patients and their relationship to chronic inflammatory response and oxidative stress.

## Materials and methods

### Study design and participants

This research was a single-center case-control investigation. The case group consisted of 100 POI patients who were admitted to our hospital between January 2020 and December 2022, whereas the control group consisted of 100 healthy individuals who were underwent physical examination at our hospital over the same time frame. This study was reviewed and approved by the ethics committee of First Hospital of Putian City (Approval number: Hospital Ethics 20221220). Due to the retrospective nature of the study, the requirement for written informed consent was waived by the ethics committee. All data were anonymized prior to analysis. All procedures were conducted in accordance with the Declaration of Helsinki (2013 revision).

### Inclusion and exclusion criteria

The diagnostic, inclusion, and exclusion criteria for POI patients were described below: (1) Diagnostic Criteria. The study group was clinically diagnosed with POI, with age <40 years old, amenorrhea duration > 4 months, and possible symptoms such as hot flashes, sweating, irritability, insomnia, etc. Some women may also experience decreased libido and memory decline. Two consecutive serum follicle-stimulating hormone (FSH) tests conducted 1 month apart showed FSH >40 IU/L, luteinizing hormone (LH) >30 IU/L, and estradiol (E2) <73.2 pmol/L. (2) Inclusion Criteria. Meeting the above diagnostic criteria; all participants were informed of the study content and voluntarily signed the informed consent form; capable of providing informed consent and completing the study procedures; normal heart, liver, and kidney functions. (3) Exclusion criteria: having a history of hormone therapy within 3 months; congenital genital malformations; recent use of sex hormone drugs, anti-infective drugs, or antibiotics; inability to cooperate with the trial; oophorectomy; hyperandrogenism (serum testosterone >2.5 nmol/L); primary amenorrhea; previous history of uterine or ovarian dysfunction and related surgical intervention.

All control subjects fulfilled the following criteria: (1) regular menstrual cycles (24–35 days) with no history of amenorrhea or menstrual disorders; (2) serum FSH <10 IU/L and E2 within the normal range for reproductive-age women; (3) no history of autoimmune diseases, chronic inflammatory diseases, or endocrine disorders; (4) no use of hormonal contraceptives or hormone therapy within 6 months prior to enrollment; (5) no history of ovarian surgery or pelvic irradiation; (6) non-smokers with no alcohol abuse; (7) body mass index within 18.5–30.0 kg/m^2^.

### mtDNAcn determination

Approximately 4 mL of peripheral venous blood was collected from the research subjects using lithium heparin vacuum blood collection tubes. DNA was uniformly extracted in the laboratory using the DNA Blood Mini Kit from QIAGEN, Germany, following the instructions provided. DNA integrity was verified by agarose gel electrophoresis, and DNA purity was assessed spectrophotometrically (A260/A280 ratios between 1.8 and 2.0). mtDNA copy number was expressed as relative copy number using the 2^∧^(−ΔCt) method, where ΔCt = Ct(ND1) − Ct(18S rRNA), normalized to the nuclear reference gene. Real-time quantitative PCR (qPCR) was performed on a Bio-Rad CFX96 Real-Time PCR Detection System (Bio-Rad Laboratories, Hercules, CA, USA) using SYBR^®^ Green qPCR Mastermix (TaKaRa, Tokyo, Japan). ND1 was selected as the mitochondrial target gene because it is a widely used and validated mitochondrial gene for mtDNAcn quantification. 18S rRNA was used as the nuclear reference gene; although a single-copy nuclear gene would be theoretically preferable, 18S rRNA is commonly used in mtDNAcn studies and has been validated for this purpose. The primers for the target gene were: ND1(F): 5′-TACACTTTTACCCACCCA-3′, ND1(R): 5′-GTTTCTTTTCTTCTTCTT-3′; the primers for the internal reference gene were: 18S rRNA(F): 5′-CGAAAGCATTTAGGAGAAT-3′, 18S rRNA(R): 5′-CTTCTGATGTGCTGAACTGA-3′. All reactions were set up in triplicate for each sample, and a no-template control was included in each run to exclude contamination. PCR efficiencies for both ND1 and 18S rRNA primers were tested using standard curve methods; efficiencies were 98.2% for ND1 and 97.5% for 18S rRNA, both within the acceptable range of 90%–110%. qPCR was performed under the following conditions: initial denaturation at 95 °C for 5 min, followed by 35 cycles of denaturation at 95 °C for 15 s, annealing at 60 °C for 30 s, and extension at 72 °C for 30 s. A melt curve analysis was performed after each run to verify the specificity of amplification. The intra-assay coefficient of variation (CV) was 2.8%, and the inter-assay CV was 4.5%, indicating good reproducibility. According to the relative quantification method, mtDNA copy number was calculated as 2^∧^(−ΔCt), where ΔCt = Ct(18S rRNA) − Ct(ND1).

### Measurement of chronic inflammatory response and oxidative stress indicators

On the day of physical examination for the control group and the day before treatment for the study group, 3 ml of blood samples were collected from the median cubital vein in a quiet and fasting state. The samples were left at room temperature for 30 min and then centrifuged at 2500 rpm for 10 min with a radius of 10 cm. Serum supernatant was transferred to a centrifuge tube and stored at −80 °C until analysis. The rationale for selecting these oxidative stress markers was as follows: ROS was chosen as a direct measure of oxidative burden. SOD was selected as a key enzymatic antioxidant reflecting the body’s capacity to scavenge superoxide anions; and GST was chosen as a phase II detoxification enzyme involved in the conjugation of electrophilic substrates to glutathione. Together, these markers provide a comprehensive assessment of the oxidative stress status from production (ROS) to defense (SOD, GST). The levels of serum tumor necrosis factor-α (TNF-α) (Wuhan Yipu Biotechnology Co., Ltd., E-EL-H0109), interleukin-1β (IL-1β) (BiooLabs, SEA563Ga), interleukin-10 (IL-10) (Nanjing Saihongrui Biotechnology Co., Ltd., HEA056Mu03), and transforming growth factor-β (TGF-β) (Wuhan Yipu Biotechnology Co., Ltd., E-EL-0162) were determined by enzyme-linked immunosorbent assay (ELISA). The levels of serum interferon-γ (IFN-γ) (Shenzhen Xinbosun Biotechnology Co., Ltd., EHC102g.96), reactive oxygen species (ROS) (Shanghai Varan Biotechnology Co., Ltd., SU-B10725), superoxide dismutase (SOD) (Beijing Ita Biotechnology Co., Ltd., YT6473), and glutathione S-transferase (GST) (Wenzhou Kemiao Biotechnology Co., Ltd., KM091061) were determined by double antibody sandwich ELISA. All samples were analyzed in duplicate, and the mean value was used for statistical analysis. The intra-assay CVs were <8% for all assays, and the inter-assay CVs were <12%. The detection ranges and sensitivities were as follows: ROS: 0.5–10 ng/mL (sensitivity 0.2 ng/mL); SOD: 10–500 U/L (sensitivity 5 U/L); GST: 1–50 U/L (sensitivity 0.5 U/L).

We acknowledge that ROS is inherently unstable and difficult to measure reliably in serum. To minimize degradation, samples were processed immediately after collection, stored at −80 °C, and analyzed within 1 month of collection.

### General information

Record the age, body mass index, blood pressure (systolic and diastolic), parity, gravidity, age of menarche, and the presence or absence of underlying diseases (diabetes, hypertension, hyperlipidemia) of the two groups of patients.

### Statistical methods

SPSS 23.0 statistical analysis software was used to process the data. Data normality was assessed using the Shapiro–Wilk test. Normally distributed data were presented as mean ± standard deviation, and group comparisons were performed using the independent samples *t*-test. Homogeneity of variance was assessed using Levene’s test. For comparisons involving multiple groups or multiple comparisons, Bonferroni correction was applied to control the family-wise error rate. The χ^2^ test compared categorical data (e.g., underlying diseases) as counts and percentages. The association between oxidative stress markers, mtDNAcn, and chronic inflammatory response in POI patients was examined using Pearson correlation analysis. The linear regression equation and the impact of oxidative stress and chronic inflammatory response markers on mtDNAcn in POI patients were obtained using general linear regression analysis. Multicollinearity was assessed using variance inflation factor (VIF) and tolerance values; VIF <2 and tolerance >0.5 were considered acceptable. The adjusted *R*^2^ was reported to account for the number of predictors in the model. ROC curve was drawn to explore the predictive value of mtDNAcn for POI. An AUC value >0.9 indicated high predictive performance, 0.71–0.9 indicated moderate predictive performance, 0.5–0.7 indicated low predictive performance, and <0.5 indicated no predictive performance. It should be emphasized that this ROC analysis is exploratory and requires external validation in independent cohorts. A *P*-value <0.05 was considered statistically significant.

## Results

### Comparison of general data between the case group and the control group

No statistically significant differences were observed between the two groups regarding continuous variables (age, BMI, blood pressure, number of pregnancies and deliveries, age at menarche) or categorical variables (smoking, alcohol consumption) (*P* > 0.05). Hormonal profiles of the control group. All healthy control subjects had regular menstrual cycles (24–35 days) and serum FSH levels <10 IU/L, confirming normal ovarian function. No control subject reported a history of autoimmune diseases, chronic inflammatory conditions, or hormone therapy use within the preceding 6 months (Table 1).

**TABLE 1:** Comparison of general data between the case group and the control group.

Indicators		Case group (*n* = 100)	Control group (*n* = 100)	Statistical values	*P*
Age (years)		36.25 ± 3.39	36.29 ± 3.40	0.083	0.934
Body mass index (kg/m^2^)		24.80 ± 0.55	24.82 ± 0.57	0.252	0.801
Systolic blood pressure (mmHg)		129.50 ± 10.03	128.51 ± 10.04	0.698	0.486
Diastolic blood pressure (mmHg)		87.60 ± 5.04	87.59 ± 5.05	0.014	0.989
Parity (times)		0.90 ± 0.55	0.93 ± 0.56	0.382	0.703
Number of pregnancies (times)		1.08 ± 0.58	1.11 ± 0.60	0.360	0.720
Age at menarche (years)		13.52 ± 1.26	13.25 ± 1.25	1.521	0.130
Underlying diseases	Yes	12 (12.00)	10 (10.00)	0.204	0.651
	No	88 (88.00)	90 (90.00)		
Smoking	Yes	8 (8.00)	6 (6.00)	0.307	0.579
	No	92 (92.00)	94 (94.00)		
Hormone replacement therapy history	Yes	32 (32.00)	–		
	No	68 (68.00)	–		
Disease duration (months)		14.50 ± 8.30	–		

Data are presented as mean ± standard deviation or *n* (%). *P* values were calculated using independent samples *t*-test for continuous variables and χ^2^ test for categorical variables. “–”: not applicable. POI: premature ovarian insufficiency. Disease duration was defined as the time interval from symptom onset (e.g., menstrual disturbance) to formal diagnosis. Hormone replacement therapy history was defined as any use of estrogen and/or progesterone therapy prior to enrollment.

### Comparison of chronic inflammatory response, oxidative stress response indicators and mtDNAcn between the case group and the control group

In peripheral blood, the case group had higher levels of serum TNF-α, IFN-γ, IL-1β, and ROS than the control group. POI patients had lower serum IL-10, TGF-β, SOD, GST, and peripheral blood mtDNAcn (*P* < 0.05) (Table 2).

**TABLE 2:** Comparison of chronic inflammatory response, oxidative stress response indicators and mtDNAcn between the case group and the control group.

Indicators	Case group (*n* = 100)	Control group (*n* = 100)	Statistical values	*P*-value
TNF-α (ng/mL)	15.25 ± 3.39	9.29 ± 1.40	17.460	<0.001
IFN-γ (ng/mL)	25.80 ± 4.55	10.82 ± 0.57	32.668	<0.001
IL-1β (pg/mL)	70.50 ± 15.13	45.51 ± 10.04	13.762	<0.001
IL-10 (pg/mL)	30.60 ± 5.04	55.09 ± 8.55	24.675	<0.001
TGF-β (ng/mL)	1.90 ± 0.55	3.93 ± 0.56	25.863	<0.001
ROS (ng/mL)	2.88 ± 0.58	1.04 ± 0.30	28.178	<0.001
SOD (U/L)	100.52 ± 25.26	200.25 ± 30.25	25.306	<0.001
GST (U/L)	12.21 ± 1.24	20.10 ± 2.54	27.914	<0.001
MtDNAcn	235.24 ± 25.24	328.21 ± 30.25	23.598	<0.001

### The value of peripheral blood mtDNAcn in predicting POI

**FIGURE 1:**
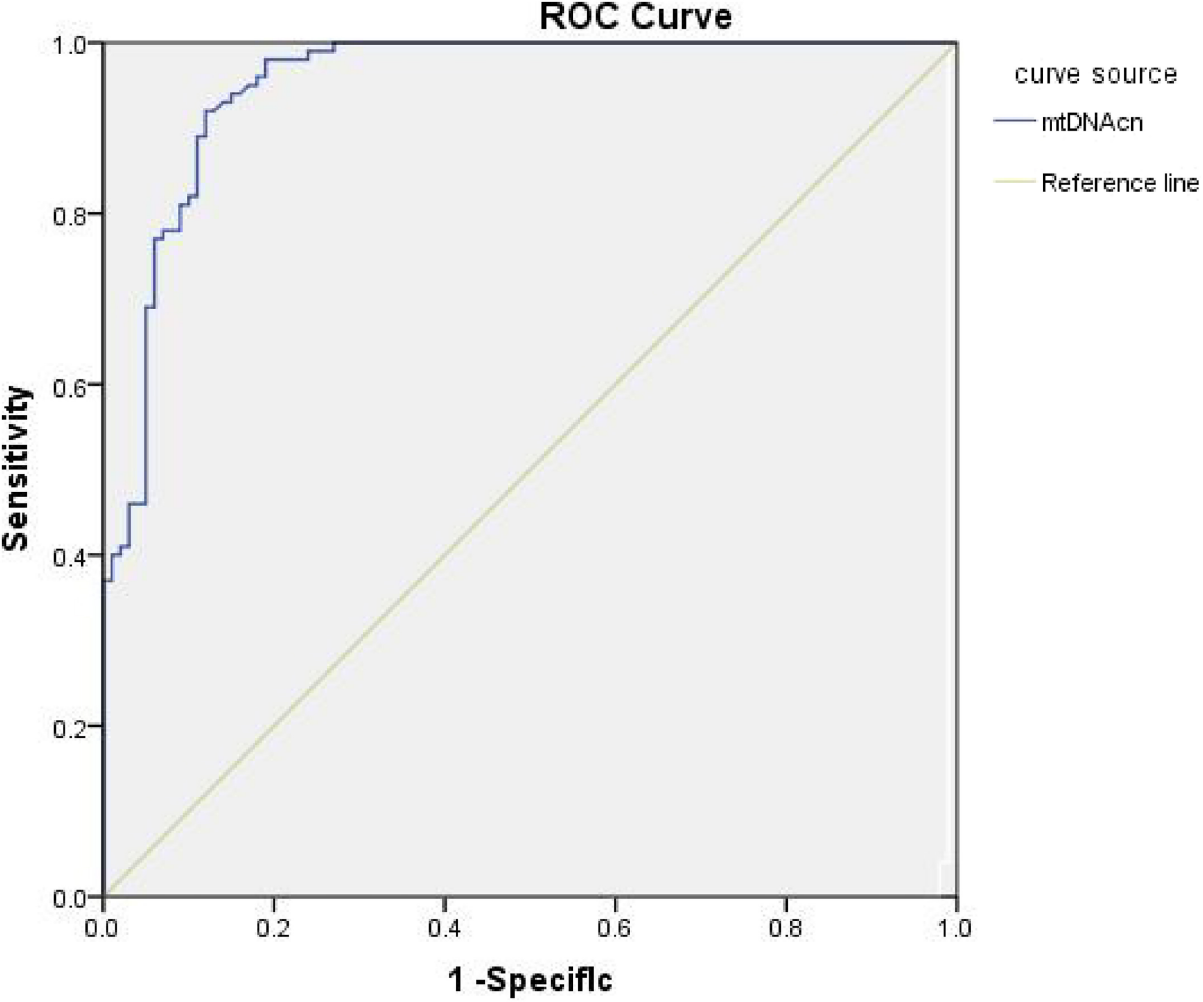
ROC curve of peripheral blood mtDNAcn for predicting POI.

The group (0 = control, 1 = case) was respectively taken as the state variable, and the level of peripheral blood mtDNAcn was taken as the test variable to draw the ROC curve. The results showed that the AUC of peripheral blood mtDNAcn in predicting POI was 0.951, with a standard error of 0.015 and a 95% CI of 0.922–0.980. The optimal cut-off value was 256.988, the sensitivity was 0.990, the specificity was 0.730, and the Youden index was 0.720 (Figure 1). However, as this was a single-center exploratory analysis without internal or external validation, the reported AUC, sensitivity, and specificity are likely optimistic and should not be interpreted as definitive diagnostic performance metrics.

### Correlation analysis

Pearson correlation analysis revealed that serum TNF-α, IFN-γ, IL-1β, and ROS were all negatively correlated with mtDNAcn (*r* < 0, *P* < 0.05); serum IL-10, TGF-β, SOD, and GST were all positively correlated with mtDNAcn (*r* > 0, *P* < 0.05) (Tables 3, 4).

**TABLE 3:** Correlation analysis between indicators of chronic inflammatory response and mtDNAcn.

Indicators	Correlation coefficient	TNF-α	IFN-γ	IL-1β	IL-10	TGF-β
mtDNAcn	*r*	−0.741	−0.800	−0.750	0.746	0.758
	*P*	<0.001	<0.001	<0.001	<0.001	<0.001

**TABLE 4:** Correlation analysis between oxidative stress response indicators and mtDNAcn.

Indicators	Correlation coefficient	ROS	SOD	GST
MtDNAcn	*r*	−0.767	0.725	0.833
	*P*	<0.001	<0.001	<0.001

### Linear regression analysis of factors associated with mtDNAcn in POI patients

Linear regression analysis showed that when mtDNAcn of POI patients was taken as the dependent variable and the variables that were correlated with mtDNAcn of POI patients in 2.3 [TNF-α (X1), IFN-γ (X2), IL-1β (X3), IL-10 (X4), TGF-β (X5), ROS (X6), SOD (X7), GST (X8)] were taken as independent variables, the multiple linear regression analysis (forced-entry method) yielded the equation: Y = 10.990–0.164 × 1−0.357 × 2−0.224 × 3 + 0.545 × 4 + 0.212 × 5−0.196 × 6 + 0.200 × 7 + 0.579 × 8. The adjusted *R*^2^ of the model was 0.892, indicating that the model explained 89.2% of the variance in mtDNAcn. The VIF of each variable was less than 2 (range: 1.239–1.507), and tolerance values were greater than 0.5 (range: 0.741–0.964), indicating that there was no significant multicollinearity among the variables. Residual analysis confirmed that the residuals were approximately normally distributed and homoscedastic. The results showed that TNF-α, IFN-γ, IL-1β, IL-10, TGF-β, ROS, SOD, and GST were all independently associated with mtDNAcn of POI patients (*P* < 0.05) (Table 5).

**TABLE 5:** Linear regression analysis of factors associated with mtDNAcn in POI patients.

Project	*B*	Standard error	Standard coefficient	*t*	*P*	95%CI	Tolerance	VIF
Constant	10.990	1.485	–	11.552	0.000	8.592 to 18.124		
TNF-α	−0.164	0.089	−0.164	−1.724	0.003	−0.239 to −0.160	0.741	1.442
IFN-γ	−0.357	0.101	−0.357	−3.546	<0.001	−0.556–0.158	0.887	1.239
IL-1β	−0.224	0.085	−0.224	−2.449	<0.001	−0.292 to −0.045	0.823	1.507
IL-10	0.545	0.038	0.545	14.519	<0.001	0.004 to 0.911	0.964	1.284
TGF-β	0.212	0.046	0.212	4.567	<0.001	0.054 to 0.608	0.915	1.355
ROS	−0.196	0.080	−0.196	−2.795	<0.001	−0.224 to −0.066	0.927	1.337
SOD	0.200	0.043	0.200	3.596	0.001	0.001 to 0.355	0.778	1.500
GST	0.579	0.108	0.579	5.370	<0.001	0.366 to 0.791	0.865	1.293

## Discussion

Ovarian aging is one of the important signs of overall aging in women, which is manifested as a decline in the number and quality of primordial oocytes, eventually leading to loss of fertility and menopause. From birth, women store a certain number of primordial oocytes in their ovaries. With aging, these oocytes will gradually mature and ovulate. Under normal circumstances, the primitive oocytes in the ovaries of women around 45 years old are almost exhausted, and it is difficult to produce enough estrogen and progesterone to maintain the periodic changes of the endometrium, which indicates that women’s ovaries are aging currently. If ovarian aging occurs before the age of 40, it will lead to POI. According to the national epidemiological study, approximately 1 to 3.8 out of every 100 women suffer from this disease, while the global prevalence is estimated to be around 1%, and the incidence rate has been increasing year by year in recent years (8, 9). Therefore, seeking biomarkers that can effectively predict the occurrence of POI is a key focus of clinical research. Previous studies have shown that there are significant differences in exosome miR-122-5p between normal ovaries and POI ovaries. The expression level of miR-122-5p in POI ovarian tissues is significantly increased, being more than three times that of normal ovarian tissues. Clinically, the content of miR-122-5p can be measured to diagnose POI (10). However, in clinical practice, the application of exosome miR-122-5p in clinical settings still faces the challenges of the inability to prepare exosomes in large quantities and the complexity and relatively high cost of obtaining natural exosomes. Therefore, it is still necessary to explore more universal biomarkers.

Current research has found that ovarian aging is closely related to mitochondrial dysfunction (11). Mitochondria are organelles widely present in human cells. Each mitochondrion contains 2 to 10 sets of mtDNA, which include 37 genes. The main function of these genes is to regulate oxidative phosphorylation and protein assembly, and they are the essential elements of mitochondria. mtDNA exists in a free form outside the cell, known as circulating cell-free mitochondrial DNA (ccf-mtDNA). Cell damage or stress can trigger the release of mitochondrial damage-associated molecular patterns (DAMPs), during which mtDNA is released into the extracellular space, forming ccf-mtDNA. Ccf-mtDNA exists in human plasma and serum and is characterized by easy accessibility and measurement. It is a biological indicator with low detection cost and simple operation. Currently, research related to mtDNAcn mainly focuses on the relationship between mtDNA and aging. Sebastian D et al. (12) pointed out that in aging organisms, changes in mitochondrial dynamics can play a significant role in cell death. Picca A et al. (13) found that the increase in mitochondrial fusion in aged rats maintains mtDNA content and helps delay aging. These studies all suggest that mitochondrial dysfunction is closely related to the occurrence and development of aging, increasing the evidence for the clinical application feasibility of mtDNAcn as a non-invasive biomarker for POI.

Some studies have pointed out that for mitochondria, when they cannot have enough mtDNA and can work properly, it will cause mitochondrial dysfunction. At this time, the production of ATP will be reduced, which can directly lead to the reduction of cell production and metabolic rate, and then affect the normal function of tissues and organs, and accelerate the aging process, especially the aging of ovaries (14). Other studies have reported that mitochondria are one of the most important organelles in oocytes. In oocytes, each mitochondrion has only one copy of mtDNA on average, so mtDNAcn represents the number of mitochondria in oocytes (15). According to the study’s findings, the case group’s mtDNAcn expression was lower than the control groups. It is evident that POI patients have far lower levels of mtDNAcn in their peripheral blood. The observed reduction in mtDNAcn may reflect a decline in oocyte quality due to low mitochondrial content and insufficient organelle biosynthesis during oocyte meiosis. This may be associated with an increased production of aneuploid oocytes and may contribute to oocyte apoptosis (16). The AUC of peripheral blood mtDNAcn in discriminating POI was 0.951, demonstrating good discriminative ability. However, it should be noted that this analysis lacked an independent validation cohort; therefore, prospective studies with external validation are required to confirm the diagnostic utility of mtDNAcn for POI. It should be noted that, due to the case-control design of this study, the observed associations between mtDNAcn and oxidative stress/inflammatory markers do not imply causation. Longitudinal and mechanistic studies are necessary to establish causal relationships.

According to research, the pathophysiology of premature ovarian failure is tightly linked to cellular immunity, and POI is an aberrant cellular immunological response induced by modifications in T lymphocyte control (17). The results of this study indicated that the levels of serum TNF-α, IFN-γ, IL-1β and ROS in the case group were higher than those in the control group, while the levels of serum IL-10, TGF-β, SOD, GST and peripheral blood mtDNAcn were lower than those in the control group. Pearson correlation analysis showed that the expression of the above indicators was all related to mtDNAcn, suggesting that the expression of peripheral blood mtDNAcn in POI patients was related to chronic inflammatory response and oxidative stress response. Through multiple linear regression analysis in this study, the equation was obtained: Y = 10.990−0.164 * 1−0.357 * 2−0.224 * 3+0.545 * 4+0.212 * 5−0.196 * 6+0.200 * 7−0.579 * 8, indicating that the indicators of chronic inflammatory response were independently associated with the mtDNAcn of POI patients. The reasons for this are as follows: First off, two crucial subsets of helper T cells are Th1 and Th2 cells. Maintaining the immune system’s regular operation and averting condition depend on the balance between Th1 and Th2 cells. Th1 cells primarily contribute to cellular immune responses by releasing cytokines including TNF-α, IFN-γ, and IL-1β, which stimulate NK cells and macrophages and improve the body’s capacity to eradicate intracellular infections. Conversely, Th2 cells primarily contribute to humoral immune responses by releasing cytokines including IL-10, TGF-β, IL-4, and IL-5, which support B cell development and proliferation as well as antibody formation, therefore offering protection against external infections.

The imbalance of Th1/Th2 cytokines caused by the increase of pro-inflammatory factors and the decrease of anti-inflammatory factors can activate the nuclear factor-kB (NF-kB) inflammatory pathway, thereby intensifying the inflammatory response of the body. In the studies by Chen GY (18) and Matzinger P et al. (19), it was pointed out that mtDNA can participate in inflammatory responses by binding to sensors such as TLR and NLRP. Among them, the TLR pathway activates subsequent inflammatory responses through NF-κB signaling, while the NLRP pathway functions through the NLRP3 inflammasome, which is basically consistent with the above analysis of this study. Persistent inflammatory stimulation can activate circulating immune cells, which in turn may induce inflammatory pathways through the activation of mtDNA, thereby generating a systemic inflammatory response. Prolonged inflammation may alter ovarian function, it may also damage the ovarian blood-ovarian barrier, ultimately resulting in ovarian tissue damage and loss of ovarian function (20, 21). Secondly, the inflammatory response can also affect the hypothalamic-pituitary-ovarian axis, leading to hormonal imbalance and abnormal ovulation; it can also influence metabolic pathways, accelerating the process of ovarian function decline through insulin resistance; and the cytokines, chemokines, nitric oxide and ROS released by inflammatory cells in circulation can further induce mitochondrial damage, as manifested by a decrease in mtDNAcn (22, 23). Thirdly, the chronic inflammatory response process can cause significant oxidative stress, leading to excessive production of ROS within the body and cells, as well as a reduction in antioxidant enzymes such as SOD and GST. At this point, ROS accumulate within cells, inducing damage to the ovarian cortex, which in turn leads to a decline in the quantity and quality of oocytes, follicular atresia, and ovarian tissue fibrosis, ultimately resulting in POI and a decrease in mtDNAcn (24, 25). Furthermore, the function of mitochondrial autophagy is compromised in low-quality oocytes, which may exacerbate mitochondrial morphological abnormalities and cause a persistent decline in mtDNAcn (26). Like this, when mtDNAcn decreases, more oxygen free radicals (like superoxide anions, hydrogen peroxide, etc.) are produced. These radicals are extremely reactive and can damage DNA, proteins, lipids, and other biological macromolecules inside cells, aggravating oxidative stress damage and creating a vicious cycle (27). Monitoring mtDNAcn changes reveals oxidative stress responses in POI. Currently, autologous mitochondrial transplantation has attracted much attention and is regarded as a feasible strategy to improve oocyte quality and a potential treatment for POI. However, this therapeutic approach still has some drawbacks, such as the difficulty in obtaining autologous stem cells, the immaturity of techniques for isolating and identifying functional mitochondria from cell cultures, and the challenge in analyzing the quantity and function of isolated mitochondria. Therefore, the effectiveness and safety of autologous mitochondrial transplantation for treating POI need further investigation.

It is important to note that peripheral blood leukocyte mtDNAcn and ovarian tissue mtDNAcn represent distinct biological compartments. While blood cell mtDNA depletion has been shown to correlate with ovarian mtDNA content in some studies, peripheral blood mtDNAcn should be considered an indirect surrogate marker rather than a direct measure of oocyte or ovarian mitochondrial function. The findings of this study do not imply direct assessment of oocyte mitochondrial content.

However, this study has several limitations. First, due to its case-control design, the observed associations between mtDNAcn and oxidative stress/inflammatory markers do not imply causation; longitudinal and mechanistic studies are needed to establish causal relationships. Second, potential confounding variables such as exercise habits, metabolic or autoimmune diseases, and detailed POI etiology were not systematically collected or adjusted for. Although key demographic factors were balanced, residual confounding from these unmeasured variables cannot be excluded. Third, although all patients were diagnosed at the time of study enrollment, the precise time interval between symptom onset and formal diagnosis was not recorded retrospectively, preventing analysis of mtDNAcn changes across different disease stages. Fourth, the ROC analysis was purely exploratory and did not employ internal validation methods (e.g., bootstrap or k-fold cross-validation) to correct for potential overfitting, nor did it have an independent external validation cohort. Therefore, the reported AUC of 0.951, sensitivity of 0.990, and specificity of 0.730 are likely subject to optimism bias and should be considered as hypothesis-generating rather than confirmatory evidence. The diagnostic utility of mtDNAcn for POI must be rigorously tested in future prospective, multicenter studies with independent validation cohorts. Fifth, the sample size was relatively modest, and all participants were recruited from a single institution, which may limit generalizability. Seventh, mtDNAcn was quantified as relative copy number using 18S rRNA as the reference gene; a single-copy nuclear gene would theoretically provide more precise normalization, and this should be considered in future studies. Future multicenter prospective studies with larger sample sizes and external validation are warranted.

## Conclusion

In conclusion, peripheral blood mtDNAcn was lower in patients with POI and was associated with inflammatory and oxidative stress markers. These findings suggest that peripheral blood mtDNAcn may serve as an indirect surrogate biomarker for POI, but direct correlations with ovarian tissue mtDNAcn and oocyte quality require further investigation. The associations observed in this study suggest that an ‘oxidation-inflammation-mtDNAcn’ axis may represent a potential link between mitochondrial dysfunction, inflammation, and POI. However, causal relationships cannot be established from this cross-sectional case-control design. The observed associations suggest that preserving mitochondrial function might be linked to reduced inflammatory and oxidative stress responses; however, causal relationships cannot be inferred from this study. These findings generate the hypothesis that protecting mitochondrial function could provide a new approach for delaying the progression of POI, which warrants testing in future mechanistic and interventional studies.

## Data Availability

The raw data supporting the conclusions of this article will be made available by the authors, without undue reservation.
